# GRcalculator: an online tool for calculating and mining dose–response data

**DOI:** 10.1186/s12885-017-3689-3

**Published:** 2017-10-24

**Authors:** Nicholas A. Clark, Marc Hafner, Michal Kouril, Elizabeth H. Williams, Jeremy L. Muhlich, Marcin Pilarczyk, Mario Niepel, Peter K. Sorger, Mario Medvedovic

**Affiliations:** 1 LINCS-BD2K DCIC, Division of Biostatistics and Bioinformatics, Department of Environmental Health, University of Cincinnati, Cincinnati, OH 45221 USA; 2 HMS LINCS Center, Laboratory of Systems Pharmacology, Department of Systems Biology, Harvard Medical School, Boston, MA 02115 USA; 3 Cincinnati Children’s Hospital Medical Center, Cincinnati, OH 45229 USA

**Keywords:** GR metrics, GR_50_, GR_max_, Data analysis, Web interface, Dose response, IC_50_, E_max_, Shiny, R package, Bioconductor, NIH LINCS program

## Abstract

**Background:**

Quantifying the response of cell lines to drugs or other perturbagens is the cornerstone of pre-clinical drug development and pharmacogenomics as well as a means to study factors that contribute to sensitivity and resistance. In dividing cells, traditional metrics derived from dose–response curves such as *IC*_*50*_, *AUC*, and *E*_*max*_, are confounded by the number of cell divisions taking place during the assay, which varies widely for biological and experimental reasons. Hafner et al. (Nat Meth 13:521–627, 2016) recently proposed an alternative way to quantify drug response, normalized growth rate (GR) inhibition, that is robust to such confounders. Adoption of the GR method is expected to improve the reproducibility of dose–response assays and the reliability of pharmacogenomic associations (Hafner et al. 500–502, 2017).

**Results:**

We describe here an interactive website (www.grcalculator.org) for calculation, analysis, and visualization of dose–response data using the GR approach and for comparison of GR and traditional metrics. Data can be user-supplied or derived from published datasets. The web tools are implemented in the form of three integrated Shiny applications (*grcalculator, grbrowser, and grtutorial)* deployed through a Shiny server. Intuitive graphical user interfaces (GUIs) allow for interactive analysis and visualization of data. The Shiny applications make use of two R packages (*shinyLi* and *GRmetrics*) specifically developed for this purpose. The *GRmetrics* R package is also available via Bioconductor and can be used for offline data analysis and visualization. Source code for the Shiny applications and associated packages (*shinyLi* and *GRmetrics*) can be accessed at www.github.com/uc-bd2k/grcalculator and www.github.com/datarail/gr_metrics.

**Conclusions:**

*GRcalculator* is a powerful, user-friendly, and free tool to facilitate analysis of dose–response data. It generates publication-ready figures and provides a unified platform for investigators to analyze dose–response data across diverse cell types and perturbagens (including drugs, biological ligands, RNAi, etc.). *GRcalculator* also provides access to data collected by the NIH LINCS Program (http://www.lincsproject.org/) and other public domain datasets. The *GRmetrics* Bioconductor package provides computationally trained users with a platform for offline analysis of dose–response data and facilitates inclusion of GR metrics calculations within existing R analysis pipelines. These tools are therefore well suited to users in academia as well as industry.

**Electronic supplementary material:**

The online version of this article (10.1186/s12885-017-3689-3) contains supplementary material, which is available to authorized users.

## Background

Measuring the relationship between the dose of a perturbagen and cellular response is a cornerstone of pre-clinical research. For simplicity, in this paper we focus specifically on drug response, but the concepts and tools discussed are applicable across studies of response to a variety of perturbagens, including small molecules, antibodies, and protein ligands. In pre-clinical pharmacology studies, response metrics are used to prioritize compounds for further analysis, investigate factors that determine drug sensitivity and resistance, and guide mechanism-of-action studies. In the case of cell-based studies using anti-cancer drugs, proliferating cells are typically exposed to drugs across a range of doses, and viable cell number (or a surrogate such as ATP level) is measured at a single subsequent point in time (often following three days of drug exposure). Relative cell count is then determined based on the ratio of the number of cells in drug-treated versus vehicle-only control wells. Data are fitted to a sigmoidal curve, which is used to compute multiple metrics of sensitivity such as the concentration of drug at which the response is half the control (*IC*_*50*_), the maximal effect at the highest dose tested (*E*_*max*_), and the area under the dose–response curve (*AUC*) [1].

However, quantification of drug dose–response using relative cell counts suffers from a fundamental flaw [2, 3]: for purely arithmetic reasons, when cells undergo fewer divisions over the course of an assay they appear more drug resistant than otherwise identical cells undergoing more divisions. The number of cell divisions that takes place over the course of an assay varies with cell density, media composition, and assay duration as well as with division rate, which is highly variable among cell lines and also differs in a systematic manner with tissue of origin and genotype [3]. The confounding effects of division rate on response as conventionally measured are sufficient to change *IC*_*50*_ values >100-fold following changes in experimental conditions that are largely arbitrary (e.g. plating density, serum concentration, assay duration etc.). Thus, dose–response curves based on relative cell count and their parameterization using *IC*_*50*_, *AUC*, and *E*_*max*_ values are fundamentally unreliable.

These issues can be addressed by measuring the sensitivity of cells to drugs on a per-division basis as computed using *GR*(*c*), the normalized growth rate inhibition value at drug concentration *c:*

$$ GR(c)={2}^{k(c)/k(0)}-1 $$

where *k*(*c*) is the growth rate of drug-treated cells and *k*(*0*) is the growth rate of untreated (or vehicle-treated) control cells. In practice, growth rates can be estimated using a fixed difference method involving the number of cells at the beginning of the treatment (*x*_*0*_) and the number of cells at the end of the assay in an untreated (or vehicle-treated) control well (*x*(*0*)) and in a drug-treated well (*x*(*c*)). The GR value is thus:

$$ GR(c)={2}^{\frac{log_2\left(x(c)/{x}_0\right)}{log_2\left(x(0)/{x}_0\right)}}-1 $$

Alternatively, if the doubling time of untreated cells, *T*_*d*_, is known from other data and is assumed to be applicable to the conditions of the dose–response experiments, the GR value can be calculated as:

$$ GR(c)={2}^{\frac{lo{g}_2\left(\frac{x(c)}{x(0)}\right)+T/{T}_d}{T/{T}_d}}-1 $$

with *T* representing assay duration.

The sign of the GR value relates directly to response phenotype: it lies between 0 and 1 in the case of partial growth inhibition, equals 0 in the case of complete cytostasis, and lies between 0 and −1 in the case of cell death. Parameterization of GR dose–response curves yields *GR*_*50*_, *GEC*_*50*_, *GR*_*max*_, *GR*_*inf*_, *GR*_*AOC*_, and Hill coefficient (*h*_*GR*_ *)* values that are largely independent of cell division rate. *GR*_*50*_, analogous to *IC*_*50*_, is the concentration at which *GR*(*c*) = *0.5*; *GEC*_*50*_, analogous to *EC*_*50*_, is the concentration at which the perturbagen has half of its maximal effect on cell growth; *GR*_*max*_ is the maximal measured effect of the perturbagen (in practice, we report the lowest GR value measured at the two highest concentrations tested); *GR*_*inf*_, analogous to *E*_*inf*_, is the maximal effect of the perturbagen as extrapolated from the GR curve rather than directly from the data (in contrast to *GR*_*max*_); *GR*_*AOC*_ (Area-Over-the-Curve is used because the GR curve can dip below zero), analogous to *AUC*, is calculated by integrating the area between the GR curve and the value 1 over a range of concentrations (in practice, we calculate *GR*_*AOC*_ directly from the GR values using the trapezoidal rule); and *h*_*GR*_ is the steepness of the sigmoidal dose–response curve. GR values can be estimated using both time-lapse and endpoint assays; in the latter case, it is necessary only to measure the number of cells in each well prior to and after drug exposure. Detailed protocols for collecting the necessary experimental data and for performing GR calculations have recently been published [4, 5].

## Implementation

**Fig. 1:**
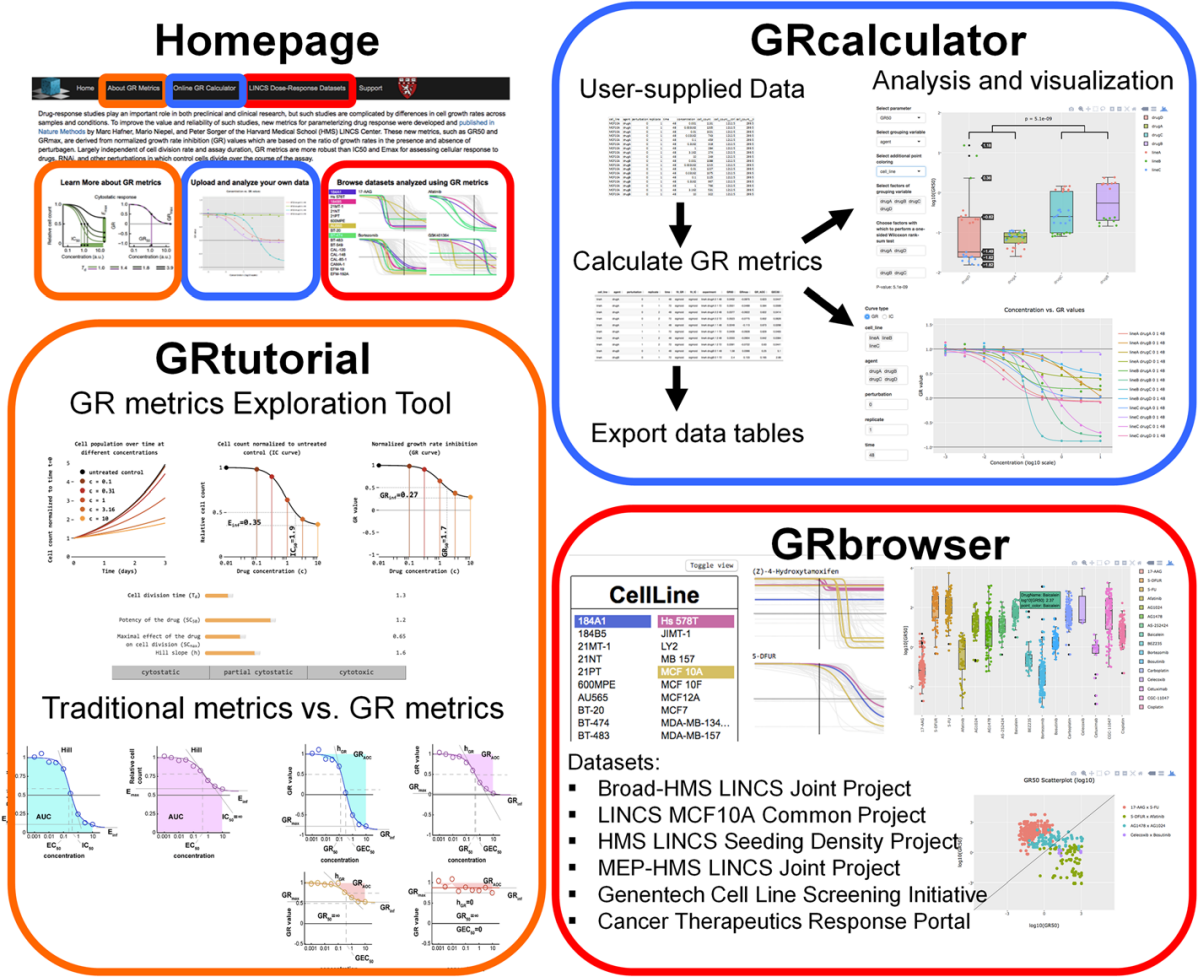
*GRcalculator* Shiny applications (*grtutorial, grcalculator, and grbrowser*) (http://www.grcalculator.org). A schematic of the *GRcalculator* homepage showing links to each of the Shiny applications that comprise it

The *GRcalculator* web tool is implemented in the form of three integrated Shiny applications (*grcalculator, grbrowser and grtutorial*) (Fig. 1) deployed via the Community Edition of Shiny Server. The Shiny instance supporting *GRcalculator* runs on a server accessible via the http://www.grcalculator.org domain.

Shiny [6] is a web application framework for R [7] that facilitates building interactive web applications using only R. It combines a seamless integration of analytical and visualization tools implemented in R with libraries of JavaScript GUI Elements. The Shiny framework also allows injection of additional JavaScript elements and modifications of underlying Cascading Style Sheets (CSS). We used the flexibility of the Shiny framework to modify some of the aspects of the default Bootstrap CSS in building GUI elements and to insert JavaScript visualization routines for displaying fitted dose–response curves. In addition to accessing *GRcalculator* via the web, the *GRcalculator* application can be launched through the R command line on a private computer or server to facilitate analysis of proprietary data. Deploying the *GRcalculator* application alone requires R version 3.3 or greater and a small number of package dependencies. Detailed instructions can be found in the “readme” document at https://github.com/uc-bd2k/grcalculator.

Two R packages developed as part of this work, *GRmetrics* and *shinyLi*, constitute the backbone of the Shiny applications deployed at http://www.grcalculator.org. The *GRmetrics* R package is used for calculating GR values [2] from user-supplied dose–response data, fitting dose–response curves to these values, and calculating GR metrics (*GR*_*50*_, *GR*_*AOC*_, *GR*_*max*_, etc.) from fitted curves. To facilitate comparison of GR and other measures of perturbagen response we have implemented tools to generate dose–response curves from relative cell count data and to calculate traditional response metrics (*IC*_*50*_, *AUC*, *E*_*max*_, etc.) from these curves. The *shinyLi* package is used for easy and intuitive grid visualization of large sets of dose–response curves within the *GRcalculator* web application.

The *grtutorial* Shiny application (accessed via the “*About GR Metrics*” link in the toolbar) provides background information about GR metrics and a description of the *GRcalculator* tools. The tutorial provides the mathematical details and scientific rationale for using GR metrics in place of traditional metrics like *IC*_*50*_ and *E*_*max*_. These points are illustrated with an interactive *Exploration Tool* (found in the “*Exploration tool*” tab) for examining the dependency of response metrics on parameters of a prototypical dose–response curve. The interactive *Exploration Tool* is implemented in the *ShinyLi* package by modifying the original JavaScript dose–response widget described in Fallahi-Sichani et al. [1]

The *grcalculator* Shiny application (accessed via the “*Online GR Calculator*” link in the toolbar) facilitates online calculation of GR values, fitting of dose–response curves (along with goodness-of-fit estimation), and calculation of GR and traditional metrics. It provides interactive visualization of GR and traditional dose–response curves, along with the points used to fit them, across multiple experimental conditions. The calculator also features interactive boxplot and scatterplot tools to explore individual GR metrics and their relation to experimental variables. The interactive graphical displays are implemented using *ggplot2*, *plotly*, and *shinyLi* packages.

The *grbrowser* Shiny application (accessed via the “*LINCS Dose–response Datasets*” section in the toolbar) facilitates interactive browsing and mining of dose–response data generated by the NIH LINCS Program as well as other published datasets. The interactive graphical displays are identical to the displays found in the *grcalculator* application, the only difference being that GR metrics are pre-computed. There are currently six datasets available on the website (see below) and we will be adding new public domain datasets to the web site as they become available.

The *GRmetrics* R package provides the key analytical functionality of the *grcalculator* application by computing GR values, fitting dose–response curves to these values, and calculating GR metrics. The *drm* function from the *drc* package is used to fit GR data to a 3-parameter logistic curve [1]. The *GRmetrics* package also contains the visualization routines found in the online version of the *GRcalculator*: GR dose–response curves along with the points used to fit them, boxplots of specific GR metrics across different experimental variables (e.g. cell lines), and scatterplots of GR metrics values (e.g. *GR*_*50*_ values for one drug against values for another drug). The package also allows for computation and visualization of traditional dose–response curves and metrics.

The *shinyLi* R package serves as the wrapper for JavaScript routines used for interactive visualization of dose–response curves and provides the grid views of dose–response curves found in the “*Dose–response Grid*” tab of the *grcalculator* and *grbrowser* applications. This is particularly useful for large dose–response datasets. The *Dose–response Grid* tool itself is an adaption of the online visualization tool previously released to visualize dose–response data described in Fallahi-Sichani et al. [1].

The suite of R scripts for calculating GR values is available as a package via Bioconductor at https://bioconductor.org/packages/GRmetrics or as MATLAB and Python scripts via the GitHub repository www.github.com/datarail/gr_metrics. For the online tool at http://www.grcalculator.org, users can upload a text file with dose–response data from their computer or provide a URL pointing to a data file (including links to Dropbox, Basecamp, or FTP sites). GR metrics will then be calculated, and the interactive visualizations described above will be produced. The resulting GR metrics datasets can then be downloaded for further analysis off-line.

## Results

*GRcalculator* integrates three basic functionalities: (1) interactive exploration of a prototypical GR dose–response model via an interactive *Exploration Tool*; (2) online and offline calculation and interactive visualization of sensitivity metrics and dose–response curves for user-supplied data; and (3) online browsing and visualization of pre-computed dose–response datasets generated from published data or data collected by the NIH LINCS Program.

### Interactive exploration tool for exploration of the GR dose–response model

**Fig. 2:**
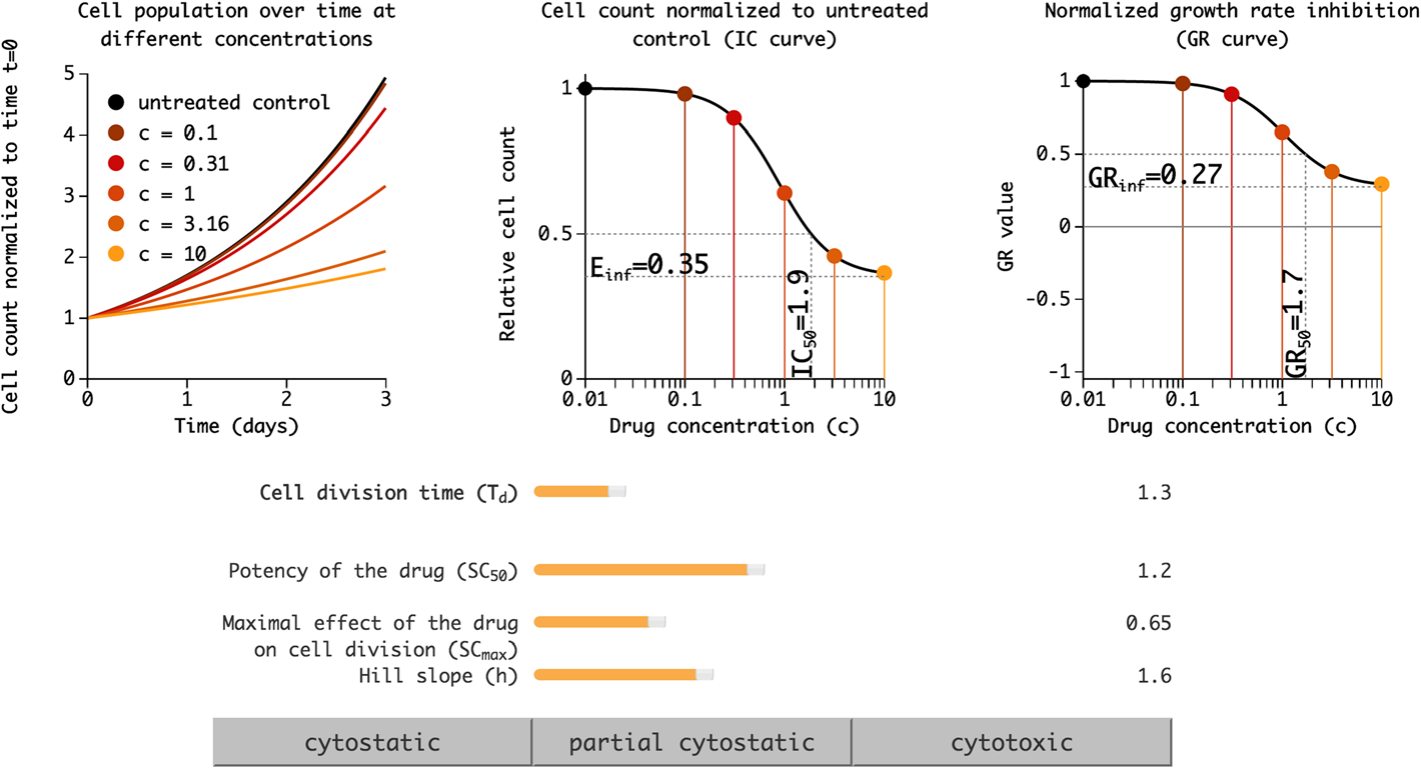
Dose–response model interactive Exploration Tool. Interactive graphs with parameters controlled by sliders show the behavior of the traditional dose–response curve (center) versus that of the normalized growth rate inhibition (GR) curve (right). Derived traditional dose–response model parameters *IC*_*50*_ and *E*_*inf*_ are displayed along with the analogous GR model parameters *GR*_*50*_ and *GR*_*inf*_. Cell population growth at different concentrations of a drug is shown over a typical 3-day assay (left). Traditional dose–response curve values and GR curve values at these concentrations are marked by similarly colored points on the center and right plots. Buttons (bottom) set parameter values to those of a typical cytostatic, partial cytostatic, or cytotoxic drug

Hafner et al. [2, 3] showed in theory and experimentally that the cell division time (*T*_*d*_) of a cell line has a confounding effect on dose–response curves computed using relative cell count. As a consequence, traditional measures of drug sensitivity (*IC*_*50*_ *, AUC, E*_*max*_) depend on division time. This is illustrated via a model exploration tool (Fig. 2) that consists of three data visualization panels with sliders that allow users to adjust the parameters of a prototypical dose–response experiment. The left panel shows cell number over time following treatment with different concentrations of drug based on cell-doubling time. The middle panel shows a dose–response curve based on relative cell count as determined at the end of the experiment (the conventional, confounded approach), and the right panel shows a GR curve for the same data. The parameters used to generate these curves can be adjusted with sliders located below the plots. Each slider represents a property of a cell line or one of the parameters of the underlying model of perturbagen response used to generate dose response data: (1) cell division time in days (*T*_*d*_), (2) the concentration at which the treatment has half its maximal effect in the model (*SC*_*50*_), (3) the maximal effect of the treatment in the model (*SC*_*max*_; values above 1 reflect a cytotoxic effect), and (4) the Hill coefficient of the equation in the model of the treatment response (*h*). A few concentration values are set by default. Buttons below the sliders can set parameter values typical of cytostatic, partially cytostatic, or cytotoxic drugs. With this tool users can see for example that the GR response curve is unaffected by changes in cell division time and that the sign of *GR*_*inf*_ determines whether a perturbagen is cytostatic (*GR*_*inf*_ *= 0*), partially cytostatic (*GR*_*inf*_ is positive), or cytotoxic (*GR*_*inf*_ is negative) for a given cell line.

### Calculating and visualizing GR metrics from user-supplied datasets

**Fig. 3:**
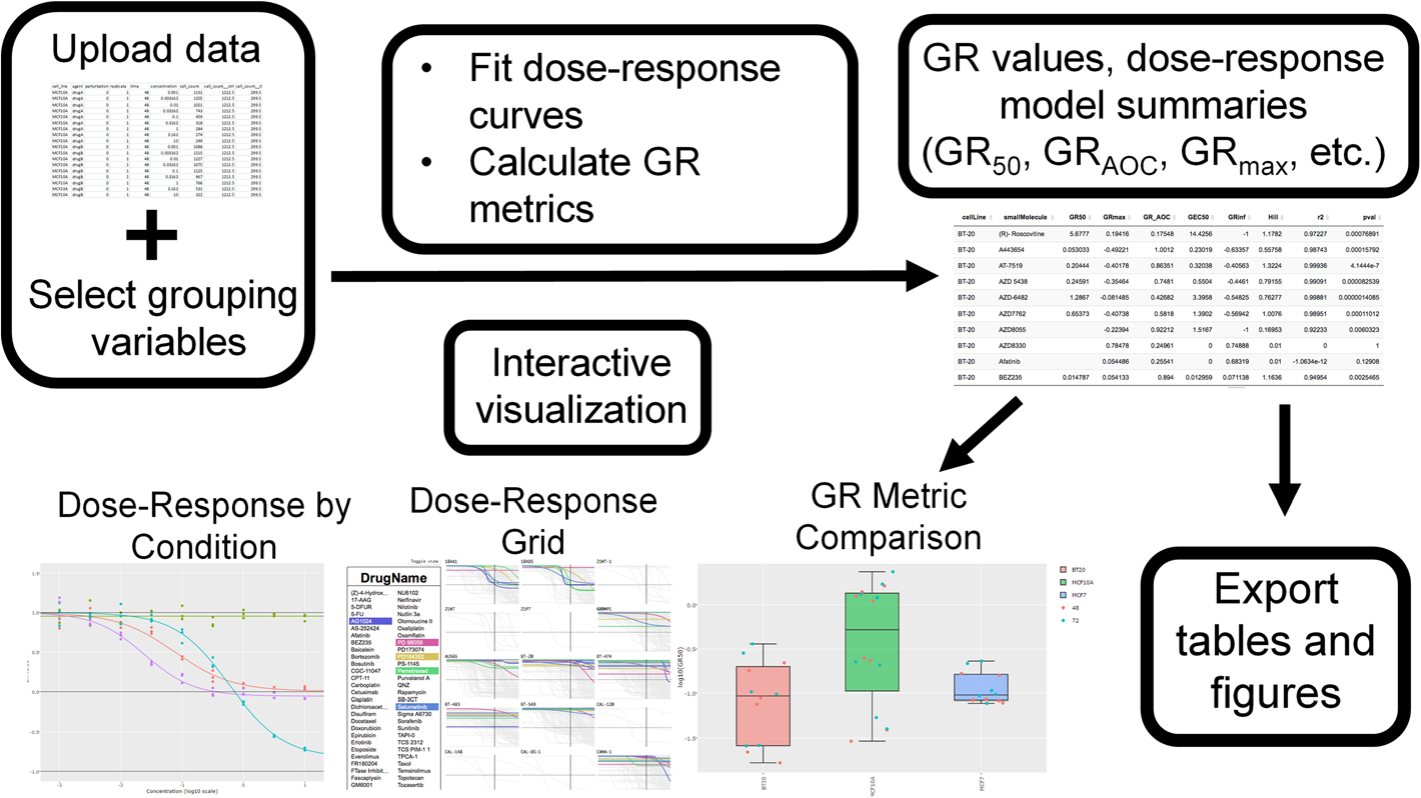
Calculating GR values and fitting dose–response curves for user-supplied data. A flowchart showing a typical *GRcalculator* workflow

The primary functionality of *GRcalculator* is to facilitate calculation and analysis of user-supplied dose–response data using the GR method (Fig. 3, upper panels). After uploading a file in the specified format or providing a link to a web-accessible file, a user chooses which “grouping variables” to use in the analysis. Each unique combination of values of the selected grouping variables defines an experimental condition. For each experimental condition, a dose–response curve is fitted across all tested concentrations in that condition. Experimental variables that are not selected as “grouping variables”, such as technical replicates, are averaged prior to GR metric calculation. For example, if the dataset contains a combination of cell lines, drugs, concentrations, and replicates, the user can select ‘cell lines’ and ‘drugs’ as grouping variables. In such case, replicates are averaged and a dose–response curve will be calculated for each pair of cell line and drug. By default, all experimental variables are considered grouping variables. Note that ‘concentration’ cannot be a grouping variable as it is considered to be the independent variable along which a dose–response curve is necessarily computed.

Running the analysis generates data tables containing the calculated GR values and derived GR metrics as well as interactive visualizations of best-fit dose–response curves and individual GR metrics organized into three additional tabs: (1) the “*Dose–response by Condition*” tab contains a plot of the GR values and the fitted dose–response curves for each experimental condition selected (Fig. 3, lower left panel); (2) the “*Dose–response Grid*” tab contains dose–response curves organized into a grid of plots defined by one of the grouping variables (Fig. 3, lower middle panel): in the example above, if the user chooses ‘drugs’ for the plot grid, each plot in the grid will contain the dose–response curves of all cell lines for a given drug; and (3) the “*GR Metric Comparison*” tab displays interactive boxplots and scatterplots of user-selected response metrics in which data points can be collapsed across multiple conditions or colored by grouping variables (Fig. 3, lower right panel). The user may also compare the underlying distributions between two box plots or groups of box plots for a particular metric using the nonparametric Wilcoxon rank-sum test. All plots are interactive: the user can zoom in and display the underlying numeric values. All data tables and plots created can be downloaded for offline analysis. A step-by-step guide to *GRcalculator* is provided in Additional file 1 and in the *GRcalculator* tutorial at http://www.grcalculator.org/grcalculator/example.html.

**Fig. 4:**
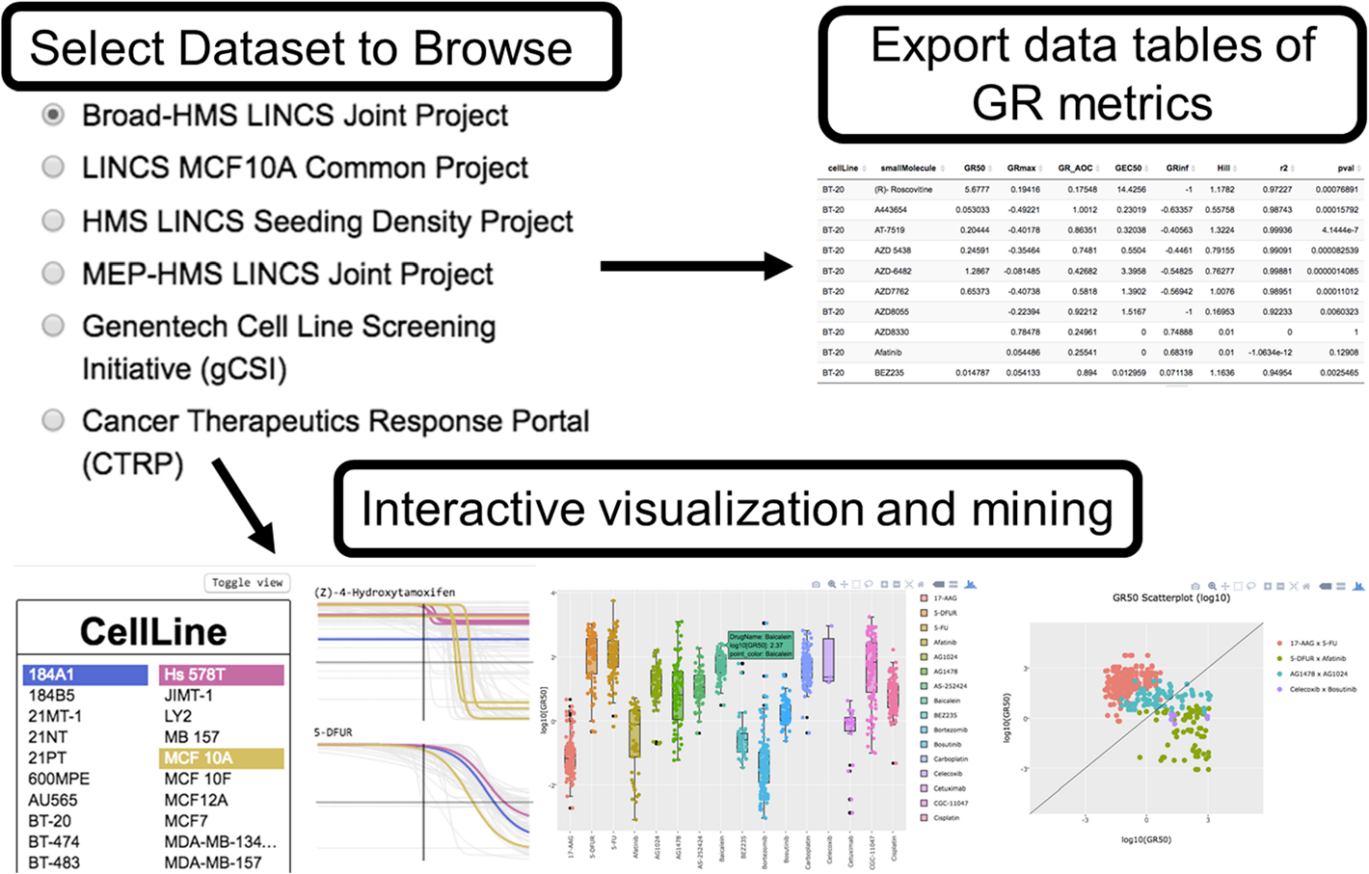
Mining LINCS and published datasets. A flowchart showing a typical *GRbrowser* workflow

The *grbrowser* application provides the same functionality as the *grcalculator* application with respect to data analysis and visualization of GR metrics, but it is specifically used for pre-loaded, publicly available datasets (Fig. 4). At the time of publication, the application contains the six datasets described below.

### Datasets available for mining

*Broad-HMS LINCS Joint Project* presents information on the responses of 6 breast cancer and nonmalignant breast epithelial cell lines to 107 different small molecule inhibitors. Cell count was measured 72 h after exposure of cells to each drug at 6 different concentrations. For further information about the experimental protocol and to download the raw data, please visit the HMS LINCS Database (http://lincs.hms.harvard.edu/db/; datasets #20245 to #20251). These data were collected in parallel with L1000 transcript profiling data as recently described [8], allowing cellular phenotype and expression state to be compared across many conditions.

*LINCS MCF10A Common Project* presents data on the response of the nonmalignant MCF10A breast epithelial cell line at 72 h to 8 small molecule drugs across a 9-point dose range. The data were collected independently by five different LINCS Data and Signature Generation Centers as a means to investigate the reproducibility and accuracy of drug dose–response data. Depending on the Center, cell number was determined either by direct counting using a microscope or by using the CellTiter-Glo assay (Promega) to measure ATP levels, a surrogate for direct cell counting.

*HMS LINCS Seeding Density Project* [2] presents the density- and context-dependent sensitivities of 6 breast cancer cell lines plated at six different densities. Cells were treated at each density with one of 12 drugs across a 9-point dose range, and viable cell number was determined at 72 h by direct counting using a microscope. For further information about the assay, please visit the HMS LINCS Database (http://lincs.hms.harvard.edu/db/; datasets #20256 and #20257).

*MEP-HMS LINCS Joint Project* presents the responses of a panel of 73 breast cancer cell lines treated with 107 small molecule and antibody perturbagens assayed by CellTiter-Glo at 72 h across a 9-point dose range. A subset of these data were described in Heiser et al. [9] and Deamen et al. [10], and re-analyzed using GR metrics in Hafner et al. [11].

*Genentech Cell Line Screening Initiative (gCSI)* [12], a large-scale drug sensitivity dataset produced by Genentech, contains data on the responsiveness of ~400 cancer cell lines from 23 tissues to 16 anti-cancer drugs. The original publication reported traditional drug response metrics based on relative cell count and we computed the GR metrics using cell doubling times available in the *gCSI* dataset [13]. Both types of metrics are presented here (with *IC*_*50*_ and *GR*_*50*_ values capped at 31 μM) along with data on the mutation status of key cancer-related genes, as reported by the Cancer Cell Line Encyclopedia (CCLE). Because of the care with which *gCSI* data were collected, this is a particularly valuable dataset for comparing GR and traditional response metrics.

*Cancer Therapeutics Response Portal (CTRP)*, described in Rees et al. [14], is a large-scale dose–response dataset created at the Broad Institute of Harvard and MIT. The data were analyzed using traditional drug response metrics based on relative cell count and we have attempted to infer GR values. To accomplish this, we estimated division times for all cell lines using gemcitabine response in the gCSI dataset [12] as a fiducial. We discarded data for cell lines for which the response to gemcitabine was weak, noisy, or missing in the gCSI dataset, resulting in GR metrics for 146 cell lines. For more details about this calculation, see Hafner et al. [3]. Because cell division times were inferred rather than measured in the CTRP data, GR values are less accurate than for the five datasets listed above.

### *GRmetrics* bioconductor package (https://bioconductor.org/packages/GRmetrics/)

**Fig. 5:**
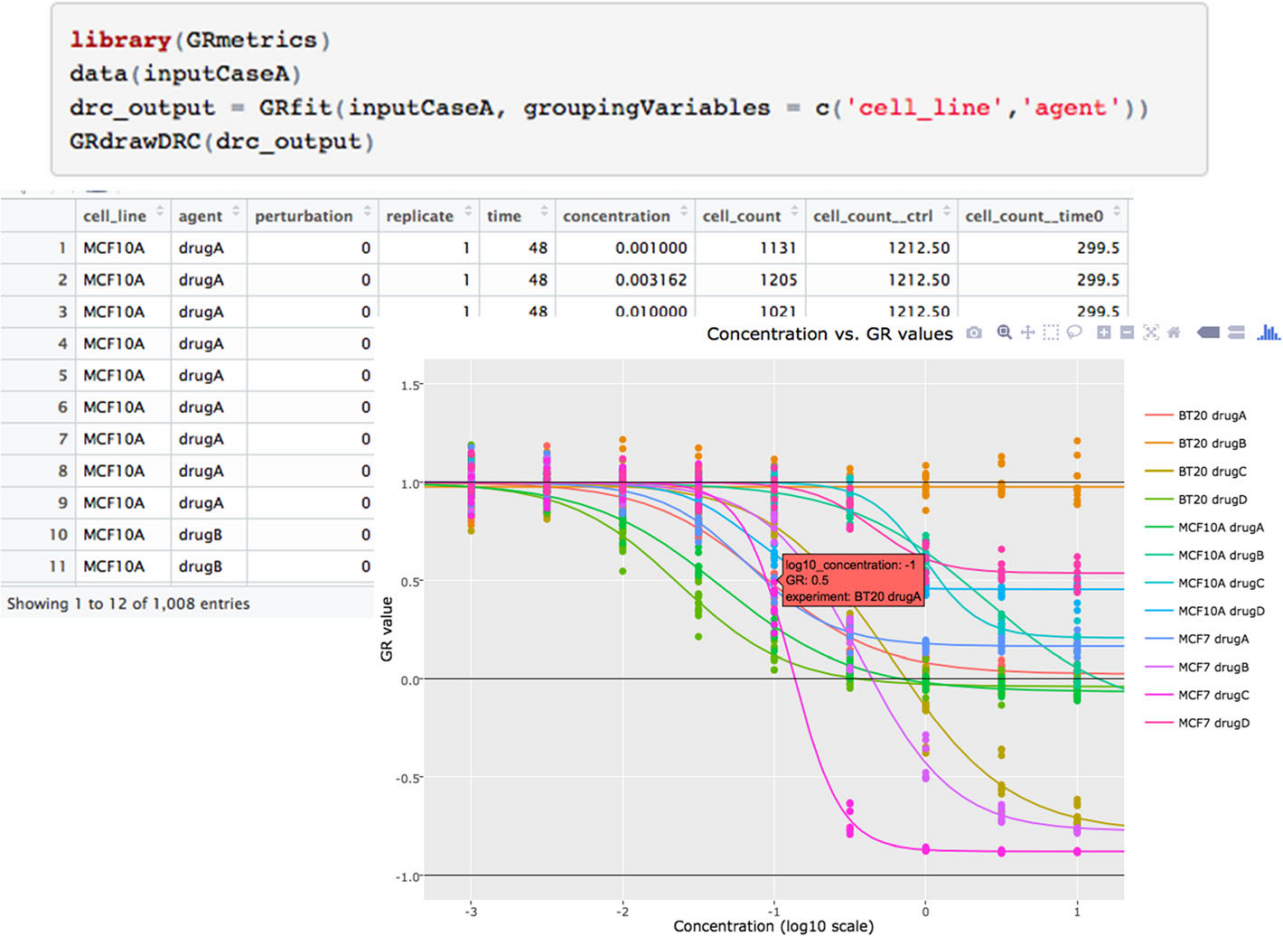
*GRmetrics* R package. Sample code and output showing generation of an interactive visualization of GR dose–response curves using the *GRmetrics* R package

The *GRmetrics* R package has two primary functions: (i) to perform the calculations needed for estimation of GR metrics (as well as traditional metrics) online via the *grcalculator* Shiny application and (ii) to enable offline GR analysis of datasets in R. The offline package provides the same visualization tools available online via *grcalculator* except for dose–response grid views. Users experienced in R or concerned about data confidentiality may prefer using the offline tool. Fig. 5 shows how data can be analyzed and visualized interactively using only a few lines of user-edited R code. The Bioconductor website for the package contains installation instructions as well as a PDF reference manual and an HTML vignette with usage notes and example code for each of the functions in the package.

### Using the *grbrowser* to explore pharmacogenomic associations

**Fig. 6:**
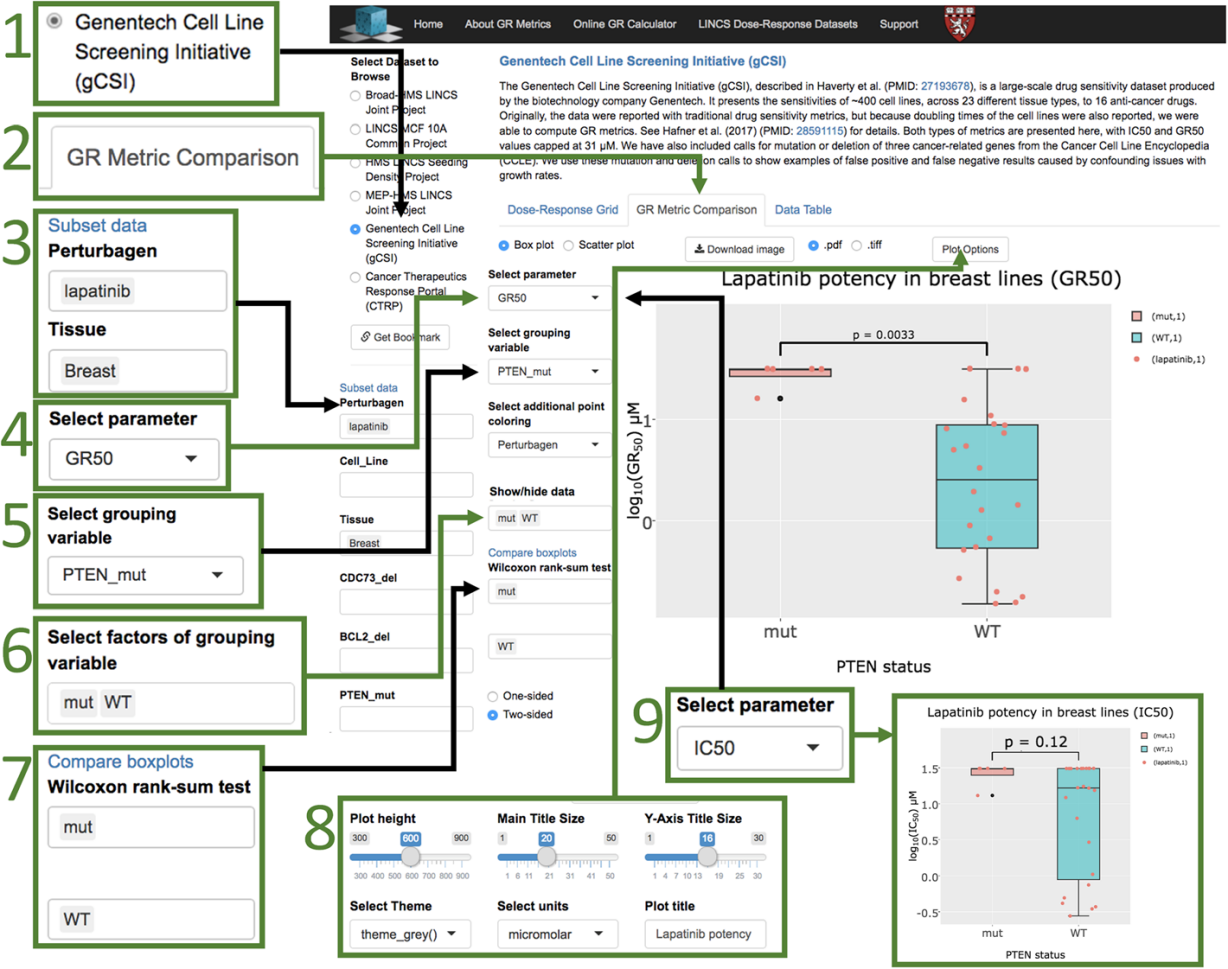
*grbrowser* use-case with gCSI data. An example use-case of the *grbrowser* with the *gCSI* dataset, reproducing a result from Hafner et al. [3]. Steps show how to use the *grbrowser* to filter the dataset to breast cancer cell lines treated with lapatinib and compare the sensitivity of wild-type PTEN cell lines with that of mutant PTEN cell lines using *GR*_*50*_ and *IC*_*50*_. In this case, use of the *GR*_*50*_ produces a known result (*p*-value 0.0033), that PTEN loss-of-function mutations mediate resistance to lapatinib in breast cancer cells, which *IC*_*50*_ fails to produce at a statistically significant level (p-value 0.12) because of large differences in growth rates between the wild-type and mutant cell lines. *p*-values were calculated using a two-sided Wilcoxon rank-sum test. *IC*_*50*_ and *GR*_*50*_ values were capped at 31 μM

By reanalyzing data from the *Genentech Cell Line Screening Initiative (gCSI)* we recently established that use of GR metrics improves the quality of pharmacogenomics associations [3]. For example, in the case of PTEN loss-of-function mutations that mediate resistance to lapatinib in breast cancer cells, we find that the *gCSI* data capture the difference when drug sensitivity is measured by *GR*_*50*_ values but not by *IC*_*50*_ values. The discrepancy arises because wild-type cell lines have a significantly slower growth rate than PTEN mutant cells, artificially increasing *IC*_*50*_ values. In Fig. 6 we illustrate how this type of comparison can performed in the *grbrowser*. In Step 1, a data set, in this case the recomputed *gCSI* dose–response metrics, is selected along with the *GR Metric Comparison* tab (Step 2). The data can be filtered by available metadata; for the *gCSI* data, a relevant perturbagen and tissue type is selected from a set of available options in a drop-down list (the drug lapatinib in breast cancer cells – note that multiple values can be selected; Step 3) along with a response metric (*GR*_*50*_ in this case) is chosen from a list of common traditional dose–response metrics and analogous GR metrics (Step 4). The *Select grouping variable* drop-down box determines the variable by which data will be separated in multiple groups; in this case, the variable is PTEN status (Step 5). The *Show/hide data* field makes it possible to add or subtract values for the grouping variable (Step 6); in the case of PTEN status this is mutant, wild-type, and NA (no data on PTEN status) but in the case of tissue type for this data, it would be a list of 23 possibilities (on the unfiltered data). The *grbrowser* then displays box plots representing the range in the response metric, in this case *GR*_*50*_ value in μM, for the PTEN wild-type and mutant grouping variables. The distributions can be compared by using a two-sided Wilcoxon rank-sum test, a robust t-test alternative; the resulting *p*-value is displayed on the graph (Step 7). Various features of the plot (titles, font sizes, etc.) can be adjusted (Step 8) to generate a publication-read figure in vector (.pdf) or bitmap format (.tiff). We see by the *GR*_*50*_ metric that PTEN mutant and wild-type breast cancer cells exhibit a highly significant difference (*p* = 0.0033) in sensitivity to lapatinib treatment, which is not found by *IC*_*50*_ value (*p* = 0.12; Step 9).

As currently constructed, the *grbrowser* makes it possible to explore internal datasets based on previously established grouping variables, but it is not yet a data discovery tool for simultaneously computing over dose–response metrics and genetic features. We plan to add features that allow users to upload and analyze previously computed dose–response metrics datasets (e.g. from *grcalculator* or *GRmetrics* R package output). This would also allow users to annotate existing datasets, for example adding additional information on tissue type or mutational status of genes as we did with PTEN in this example. As it stands now, the *grbrowser* provides a small number of manually curated dose–response datasets for viewing and mining. However, because the GR metrics methodology harmonizes dose–response data from disparate sources that previously would have been confounded by differences in the number of cell divisions taking place during an assay, there is an opportunity for researchers to combine dose–response datasets that previously would not have been compatible.

## Discussion

Tools commonly used to analyze dose–response data (such as Prism) are not yet capable of computing GR metrics, which is the best method available for eliminating biases in measuring perturbagen dose–response in proliferating cells. Use of GR metrics makes it possible to reliably compare data on drug potency and efficacy across cell lines having different underlying rates of division, assayed for different lengths of time, or growing at different rates due to changes in culture conditions. Given properly processed data, the online and offline tools described here calculate GR values, fit these values to a sigmoidal curve, evaluate the significance of the sigmoidal fit using an F-test, and yield GR metrics. To avoid contaminating dose–response datasets with low reliability values extrapolated from poor fits, non-significant curve fits are replaced by a flat line, and response metrics are set to default values. After calculating the sensitivity metrics, users can quickly and simply visualize results, perform basic analyses, and produce publication-ready figures. Offline R-based *GRcalculator* tools are designed for computationally sophisticated users and those with proprietary data. The choice of R [7] for online and offline GR calculations facilitates re-use of existing tools for fitting dose–response curves [15] and has enabled creation of a *GRmetrics* Bioconductor [16] package to facilitate integration of GR metrics within R analytical workflows. For example, combining *GRmetrics* with the *PharmacoGx* [17] Bioconductor package facilitates the use of GR metrics in pharmacogenomics analyses.

Reproducibility has become a major concern in contemporary biomedical research and the use of GR metrics increases reproducibility by correcting for factors that are often poorly controlled in large-scale studies involving many cell lines. These factors include plating density and number of cell divisions [3]. Standardization of assay methodology [4] and of computational tools and pipelines for converting raw data into final results [5] are essential for making data acquisition and analysis consistent across experiments; the *GRcalculator* meets these requirement and helps to avoid data processing artefacts. *GRcalculator* also serves as a repository for large-scale dose–response datasets that have been analyzed using the GR approach, thereby providing a reliable and reusable set of information for the community. The number of such datasets is currently small (primarily due to limitations in existing experimental data), but future dose–response data collected by the NIH LINCS Program will be released in *GRcalculator* and we anticipate that this will also be true of other efforts focused on characterizing the responses of cells to perturbation. We anticipate further development of the GR method and of other ways of calculating drug response over time [2, 18] and will therefore update the *GRcalculator* website as needed.

## Conclusions

GR metrics facilitate reliable and reproducible comparisons of drug efficacy and potency across cell lines having different cell division rates. GR metrics can eliminate false positive and false negative findings arising from the use of traditional *IC*_*50*_, *AUC*, or *E*_*max*_ values. The online and offline *GRcalculator* tools described in this paper facilitate adoption of GR metrics for the analysis of dose–response data by a wide range of users. Online *GRcalculator* tools are user-friendly and simple; they enable interactive exploration of a prototypical GR dose–response model*,* calculation and interactive visualization of user-supplied data, and online browsing and visualization of pre-computed datasets. Offline tools implemented in the *GRmetrics* Bioconductor package facilitate integration of GR metrics calculation within R analytical workflows and processing of confidential data offline.

## Availability and requirements

**Project name:** GRcalculator.

**Project home page:** http://www.grcalculator.org

**Programming languages:** R, JavaScript.

**Operating system(s):** Platform independent.

**Other requirements:** R (> = 3.3) Bioconductor 3.4 or higher.

**License:** GPL-3.

**Any restrictions to use by non-academics:** None.

## Additional file

Additional file 1: Step-by-Step GR Calculator Example. Supplementary document describing step by step example of using *GRcalculator* (PDF 1256 kb)

## Abbreviations

AUC: area under the traditional dose–response curve
EC_50_: the concentration of drug when it produces half of its maximal effect (*E*_*inf*_) extrapolated from the traditional dose–response curve. In the fitting procedure, *EC*_*50*_ is constrained to lie within two orders of magnitude of the highest and lowest tested drug concentration range.
E_inf_: drug efficacy extrapolated to an infinitely high drug concentration as determined from the asymptote of a traditional dose–response curve. For dose–response curves that reach a plateau at the highest tested concentrations, the value *E*_*inf*_ is similar to *E*_*max*_.
E_max_: the traditional metric of efficacy; the number of cells in the well treated at the highest concentration divided by the number of cells in a vehicle-treated control well.
GEC_50_: analogous to *EC*_*50*_, the concentration of drug at half-maximal effect. *GEC*_*50*_ is relevant for drugs having poor efficacy for which the response does not reach GR values below 0.5. In the fitting procedure, *GEC*_*50*_ is constrained to lie within two orders of magnitude of the highest and lowest tested drug concentration range.
GR/GR curve: the normalized growth rate inhibition values and the associated dose–response curve. By contrast, we refer to the curve based on relative cell count as the “traditional” dose–response curve or occasionally the “IC curve” as in *IC*_*50*_.
GR_50_: analogous to *IC*_*50*_, the primary GR metric for drug potency; the concentration, *c*, of a drug at which *GR(c) = 0.5*. If the value for *GR*_*inf*_ (see below) is above 0.5, *GR*_*50*_ cannot be defined and we set its value to +∞.
GR_AOC_: the integrated effect of the drug across a range of concentrations as estimated from the “area over the curve” (for the GR dose–response curve). A value of 0 means no effect of the drug across the full dose–response range. *GR*_*AOC*_ can only be compared across drugs or cell lines when the dose range is the same.
GR_inf_: the drug efficacy extrapolated to an infinitely high drug concentration as determined from the asymptote of the GR dose–response curve; *GR*_*inf*_≡*GR*(*c* → ∞). For dose–response curves that reach a plateau at the highest tested concentrations, the value *GR*_*inf*_ is similar to *GR*_*max*_.
GR_max_: the primary GR metric for drug efficacy; the GR value at the highest tested dose of the drug. *GR*_*max*_ lies between −1 and 1; negative values correspond to a cytotoxic response (i.e. cell death), a value of 0 corresponds to a fully cytostatic response (no increase in cell number), and positive values less than one correspond to partial growth inhibition.
h: the Hill coefficient of the traditional dose–response curve; it reflects the steepness of the curve. We constrain its value between 0.1 and 5.
h_GR_: the Hill coefficient of the GR dose–response curve; it reflects the steepness of the curve. We constrain its value between 0.1 and 5.
IC_50_: the traditional metric of potency; the concentration of a drug at which the number of treated cells is half the number of untreated or vehicle-treated control cells. If the value for *E*_*inf*_ (see below) is above 0.5, *IC*_*50*_ cannot be defined and we set its value to +∞.
LINCS: Library of Integrated Network-Based Cellular Signatures, a multi-center NIH Common Fund Program.
SC_50_: the concentration at which the treatment effect is half its maximal in the theoretical model of drug response.
SC_max_: the maximal effect of the treatment in the theoretical model of drug response; values above 1 reflect a cytotoxic effect.
T_d_: cell division time in days.
