# Supplementary material for: GRcalculator: an online tool for calculating and mining dose–response data
Source: BMC Cancer. 2017 Oct 24;17:698. doi: 10.1186/s12885-017-3689-3 (PMC5655815; doi:10.1186/s12885-017-3689-3)
Supplement: Additional file 1: — Step-by-Step GR Calculator Example. Supplementary document describing step by step example of using GRcalculator (PDF 1256 kb) [file 12885_2017_3689_MOESM1_ESM.pdf]

Step-by-Step GR Calculator Example

To illustrate the utility and convenience of the GR Calculator and show a real-world example of its usage, we will walk through a quick analysis of the response of breast cancer cell lines to taxol as measured by Heiser et al. (2012)[1]. To access the GR

Fig 1: grcalculator.org homepage

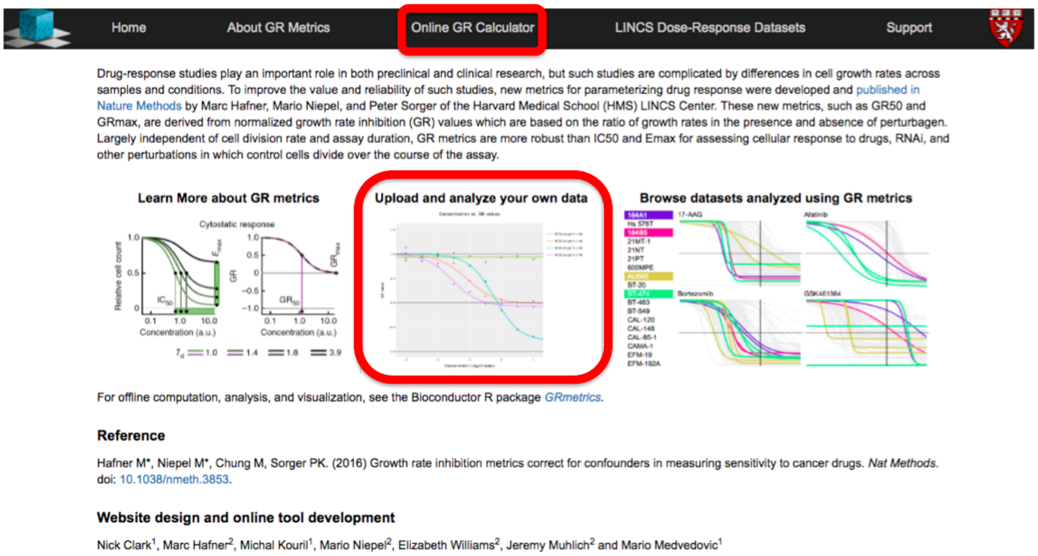

Calculator from the grcalculator.org homepage, click “Online GR Calculator” in the top banner or click the middle image (“Upload and analyze your own data”). This will bring you to the GR Calculator application where you can input and analyze drug response datasets.

In the middle of the page you will find instructions on how to format input files. There are two acceptable formats described. The data for this example is in Format A. The required columns for this format are listed in the instructions: the concentration of the drug, the cell count at the time of treatment, and cell counts of the treated and control samples at the end of the treatment. These columns must be named “concentration”, “cell_count__time0”, “cell_count”, and “cell_count__ctrl”, respectively. Any additional column, such as drug, cell line, or replicate, is considered a grouping variable. You may use either a comma-separated file (.csv) or a tab-delimited file (.tsv) for input. The dataset used in this example can be found here: https://raw.githubusercontent.com/uc-bd2k/grcalculator/master/resources/Heiser_al_GRmetrics_wSubType_Taxol.tsv.

To get started, click “Open data file” and select “Tab” as a separator (our file is tab-delimited). You may either paste the URL from above into the text box and click the “Fetch Data” button, or if you downloaded the data, you may click the “Browse…” button and select the file you just downloaded. This dataset contains experimental data of the response of breast cancer cell lines to the microtubule stabilizer taxol and is a subset of the public domain dataset http://dx.doi.org/10.7303/syn8094063.1 generated by Heiser et al. (2012)[1]. The data file also contains the clinical subtypes (HER2amp for

HER2 amplified, HR+ for hormone receptor positive, NM for non-malignant, TNBC for Triple negative breast cancer, and Unknown for Unknown subtype) and transcriptional subtypes (Basal, Luminal, etc.) for each cell line. Taxol has been shown to be effective in

Fig 2: The GR Calculator application

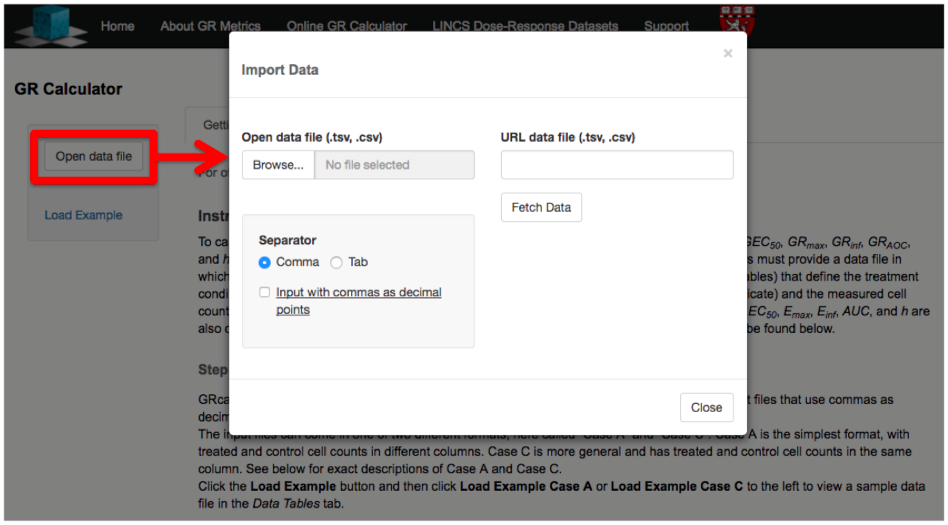

patients with either HER2 positive or TNBC cancers[3,4,5], so we would expect it to have a stronger effect in the HER2 amplified and TNBC cell lines than in the ones of other subtypes. This difference should be reflected by lower values of the sensitivity metrics IC_{50} and GR_{50} for the HER2amp and TNBC cell lines. After analyzing the data, we will visualize these differences using the tools developed here.

Now, once the dataset is loaded (Fig. 3), remove “BiolReplicate” from the “Select grouping variables” box by clicking on it and pressing the “delete” key. Removing

Fig 3: Response data for taxol loaded in the GR Calculator

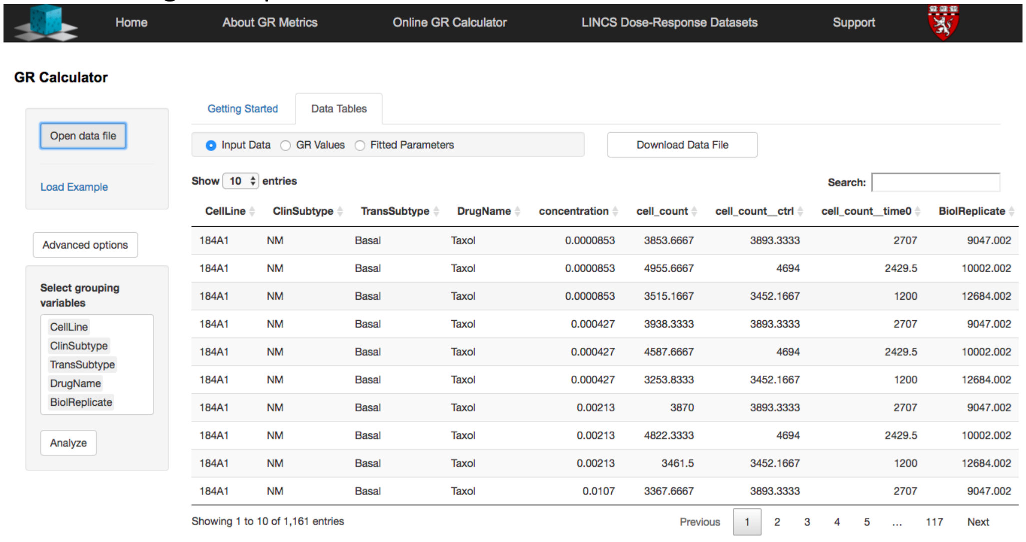

grouping variables tells the calculator to average over those unselected variables; here we decided to average the biological replicates. Click the “Analyze” button to perform the curve fitting and calculate the GR and traditional metrics. This populates the “GR Values” and “Fitted Parameters” data tables and creates three new tabs: “Dose-Response by Condition”, “Dose-Response Grid”, and “GR Metric Comparison”.

The “Dose-Response by Condition” tab contains the fitted GR curves for each condition (Fig. 4). You may select any combination of values for each grouping variable to choose which curves you would like to see. The x-axis is the log (base 10) of concentration; the y-axis is either the GR value or relative cell count, depending on whether you select GR or IC as “curve type”. In the figure, we have selected “HER2amp” and “TNBC” clinical subtypes, “Basal A” transcriptional type, and of course “Taxol” as the drug. Leaving the “CellLine” box empty automatically selects all cell lines.
For datasets with many conditions, the “Dose-Response Grid” tab allows to quickly view many curves at once in a grid format (Fig. 5). You can choose between the GR and traditional (IC) curves. The first drop-down menu at the top, “Choose selector variable”, controls which values can be selected to highlight samples on the grid. The second selection box at the top, “Choose grid variables”, allows the user to choose which grouping variables define the grid. In the figure, we chose the combination of clinical and transcriptional subtypes to define the grid and we chose cell lines as the variable for highlighting samples; in the box on the left we picked a few specific cell lines.

Fig 4: Dose-Response By Condition tab

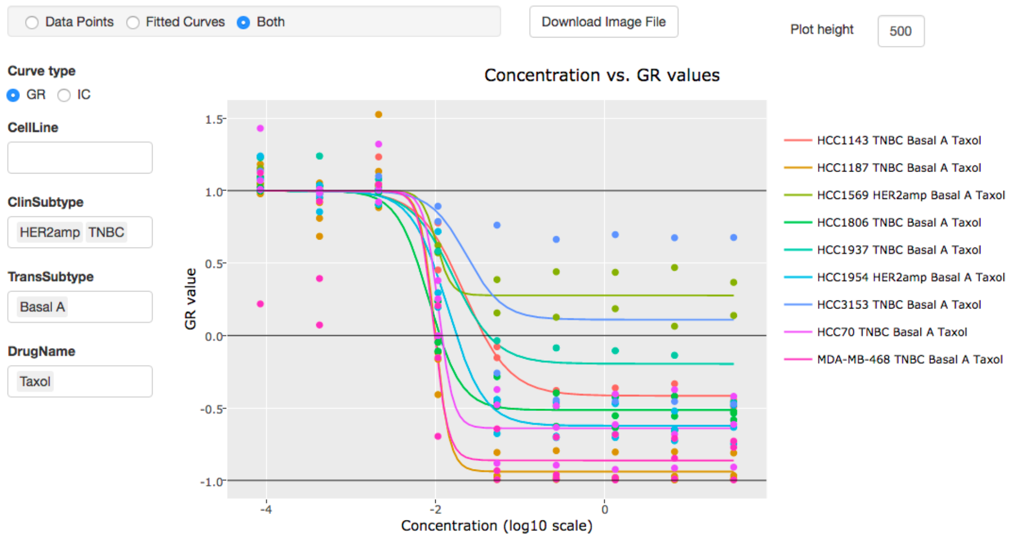

Fig 5: Dose-Response Grid tab

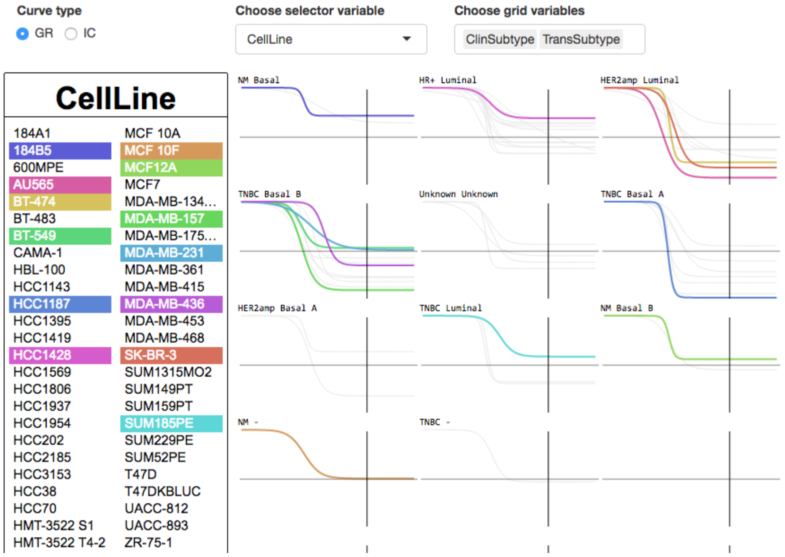

The tab “GR Metric Comparison” displays boxplots or scatter plots based on the GR or traditional metrics (Fig. 6). The boxplots can be particularly useful for comparing the potency or efficacy of a drug across many different conditions. The top selection box, “Select parameter”, allows you to choose the metric you would like to use (y-axis). The next selection box, “Select grouping variable”, allows you to specify the variable (e.g. clinical subtype) that will determine which values define the boxplots. By default up to ten boxplots are shown, but the user can select and de-select boxplots. Additionally, one may perform a Wilcoxon rank-sum test to assess if the values from one boxplot (or a set of boxplots) are significantly different from the ones from another boxplot or set of boxplots.

Fig 6: Boxplots of GR_{50} for taxol across clinical subtypes

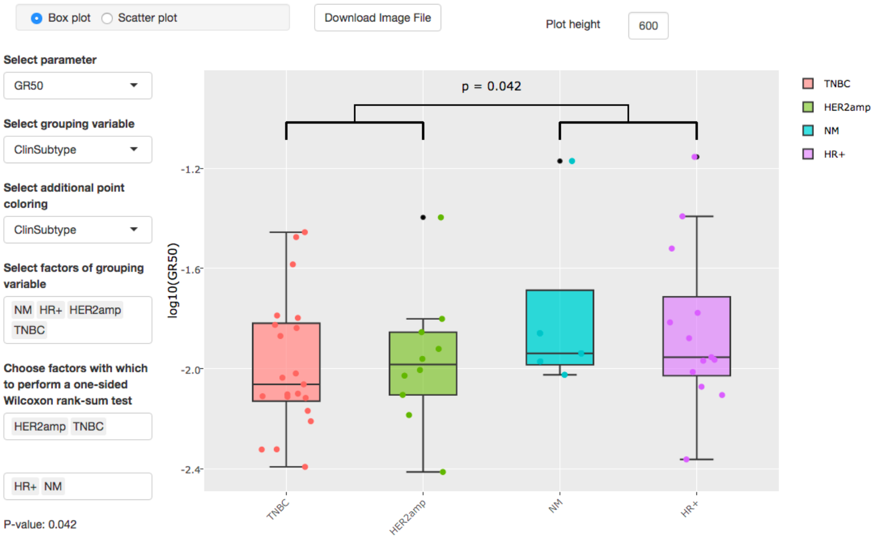

Figures 6 and 7 illustrate the GR_{50} and IC_{50} across the four clinical subtypes in the data (excluding “Unknown”). As stated before, we expect that values for the TNBC and HER2amp cell lines will be significantly less than those of the other clinical subtypes. After selecting “GR50” as our metric, we select “ClinSubtype” for the grouping variable and the point coloring, and we choose to compare “HER2amp” and “TNBC” cell lines against “HR+” and “NM” cell lines. By the p-value of 0.042, we can see that this group (“HER2amp” and “TNBC”) seems to have significantly lower GR_{50} values than the other clinical subtypes, as expected.

After verifying that taxol is most potent on HER2amp and TNBC cell lines as measured by GR_{50} values, we explore whether this result is the same using the traditional dose-response metric, IC_{50}. To do this, we simply change our parameter selection to “IC50”. As shown in Figure 7, the p-value is 0.42, indicating no significant difference in the toxicity of taxol between the two groups of clinical subtypes. From this example we see that the GR Calculator and the underlying GR value method is useful for detecting differences that can be hidden when using IC_{50} values, as traditional metrics are confounded by differences in division rates[2].

Fig 7: Boxplots of IC_{50} for taxol across clinical subtypes

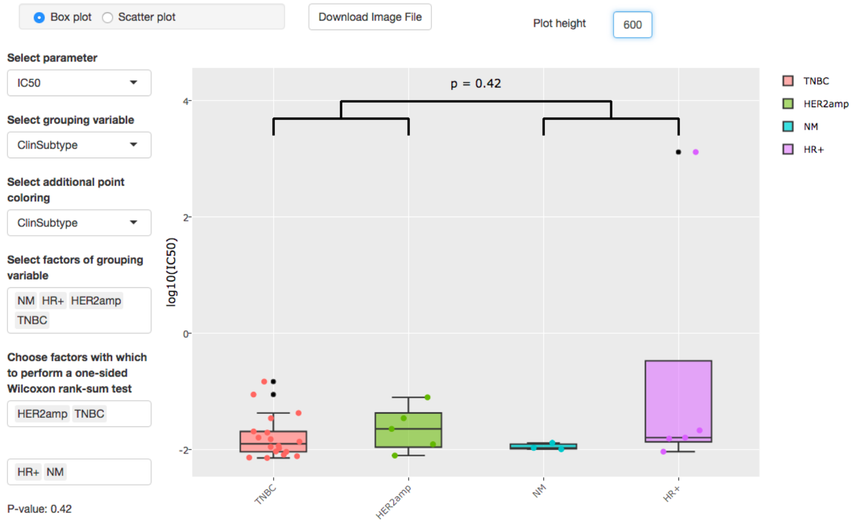

Most plots are interactive and will show extra information dynamically. For example, hovering over points and curves in the “Dose-Response by Condition” tab will display the GR value or relative cell count and corresponding concentration. In the “GR Metric Comparison” tab, hovering over a boxplot will display the maximum, minimum, and median values. The user can also hover over individual points in the boxplot and scatter plot visualizations to see detailed information. In addition, the user can adjust the plot height, zoom in, and download the figure in pdf or tiff format.

References:

1. Heiser LM, Sadanandam A, Kuo W-L, Benz SC, Goldstein TC, Ng S, Gibb WJ, Wang NJ, Ziyad S, Tong F et al: Subtype and pathway specific responses to anticancer compounds in breast cancer. Proceedings of the National Academy of Sciences 2012, 109(8):2724-2729.
2. Hafner M, Niepel M, Chung M, Sorger PK: Growth rate inhibition metrics correct for confounders in measuring sensitivity to cancer drugs. Nat Meth 2016, 13(6):521-527.
3. Blanchard Z, Paul BT, Craft B, ElShamy WM: BRCA1-IRIS inactivation overcomes paclitaxel resistance in triple negative breast cancers. Breast Cancer Research 2015, Jan 13;17:5
4. Mustacchi G, De Laurentiis M: The role of taxanes in triple-negative breast cancer: literature review. Drug Design, Development and Therapy 2015, Aug 5;9:4303-18
5. Hudis CA, Gianni L: Triple-negative breast cancer: an unmet medical need. Oncologist. 2011;16 Suppl 1:1-11
